# Extranodal natural killer/T cell lymphoma of the skeletal muscle

**DOI:** 10.1007/s00256-024-04680-w

**Published:** 2024-04-20

**Authors:** Ettore di Gaeta, Floortje Verspoor, Dilara Savci, Naomi Donner, Mario Maas, Robert Hemke

**Affiliations:** 1 Department of Radiology and Nuclear Medicine, Amsterdam University Medical Centers, Amsterdam Movement Sciences, Amsterdam, the Netherlands; 2 Department of Radiology, IRCCS Ospedale San Raffaele, Via Olgettina 60, 20133 Milan, Italy; 3 Department of Orthopedic Surgery, Amsterdam University Medical Centers, Amsterdam Movement Sciences, Amsterdam, the Netherlands; 4 Department of Pathology, Amsterdam University Medical Centers, Amsterdam, the Netherlands

**Keywords:** Musculoskeletal, Lymphoma

## Abstract

This case report highlights a case of extranodal NK/T cell lymphoma initially misdiagnosed as myositis, emphasizing the appearance on both MRI and FDG PET images. The patient presented with systemic symptoms and calf muscle swelling, prompting imaging studies that revealed diffuse muscle involvement. Despite negative myositis markers and inconclusive biopsy, post-amputation findings confirmed lymphoma with EBV positivity. The appearance in both MRI and FDG PET complicated the diagnostic process, underscoring the importance of considering lymphoma in cases of muscle-related symptoms to prevent delays in appropriate management. This case contributes to the understanding of the diagnostic challenges associated with extranodal NK/T cell lymphoma and emphasizes the significance of peripheral band-like features in imaging studies.

## Introduction

Natural killer (NK)/T cell lymphoma, a rare subset of non-Hodgkin’s lymphoma (NHL), comprises approximately 5–18% of NHL cases [1]. Predominantly, it manifests as destructive lesions localized in the nasal cavity and midline facial structures, while around one-third of instances present in areas beyond the nasal region, such as the skin, testicles, liver, spleen, and notably, infrequently involving skeletal muscle [3]. The role of Epstein-Barr virus (EBV) in the pathogenesis of extranodal NK/T-cell lymphoma is suspected, though the precise mechanistic details remain elusive [4].

Accurate diagnosis is crucial to avoid unnecessary surgical interventions. Differentiating skeletal muscle lymphoma from various neoplastic and inflammatory disorders based solely on clinical and imaging findings can be challenging, as illustrated by Chun et al. [5]. Magnetic resonance imaging (MRI) patterns often feature mass formation with homogeneous and diffuse enhancement, or abnormal muscle intensity with peripheral thick band-like enhancement. While no definitive correlation between imaging and pathological features has been established, some natural killer T-cell lymphomas exhibit peripheral band-like abnormal signal intensity, suggesting an infiltrative process.

Within the MRI context, distinguishing skeletal muscle lymphoma from other malignant soft tissue tumors hinges on specific features, including extensive segmental involvement of skeletal muscle, engagement of contiguous vessels, involvement of multiple compartments, subcutaneous stranding, and skin thickening. These characteristics are rarely encountered in other malignant muscle tumors. It is noteworthy that predominantly peripheral thick band-like enhancement or marginal septal enhancement may be observed in skeletal muscle lymphoma. This can pose a diagnostic challenge, particularly in cases featuring deep fascial enhancement that may mimic conditions necessitating urgent surgical intervention [6].

Liu et al. have reported that pathological examination of muscle biopsy samples commonly reveals a significant amount of necrosis and the regeneration of individual muscle fibers, a phenomenon not unique to skeletal muscle lymphoma but also observed in conditions such as necrotizing fasciitis and immune-mediated necrotizing myopathy (IMNM) [7].

In the present report, we show a case with an initial clinical presentation of calf muscle swelling. Ultimately, a conclusive diagnosis of NK/T cell lymphoma was achieved via postmortem examination. Additionally, we explore factors that contributed to the initial misdiagnosis of myositis and offer a review of relevant literature related to muscles affected by extra-nodal NK/T cell lymphoma.

## Case report

The patient initially presented to our hospital with a several-month history of general fatigue, night sweats, and pain in the right calf. The pain was severe enough to disturb his sleep. Simultaneously, the patient experienced a throat infection with symptoms of chills, clamminess, and excessive sweating. His throat infection was initially treated with amoxicillin and these symptoms improved. However, the right lower leg became swollen, and a noticeable decrease in strength was objectified on physical examination. When he presented to our institution’s ER, he did not have a fever but referred malaise weight loss and persistent swelling of the lower limb. To rule out deep venous thrombosis (DVT), a leg ultrasound (US) was performed, revealing non-homogeneous enlargement of all leg muscles, without any fluid collection or mass-like lesions, and associated with diffuse edema of the surrounding soft tissues.

Laboratory tests revealed an elevated erythrocyte sedimentation rate (112 mg/L) and positivity to Epstein bar virus (EBV). Over the next period of time, his condition deteriorated and his lower leg swelling progressed.

**Fig. 1:**
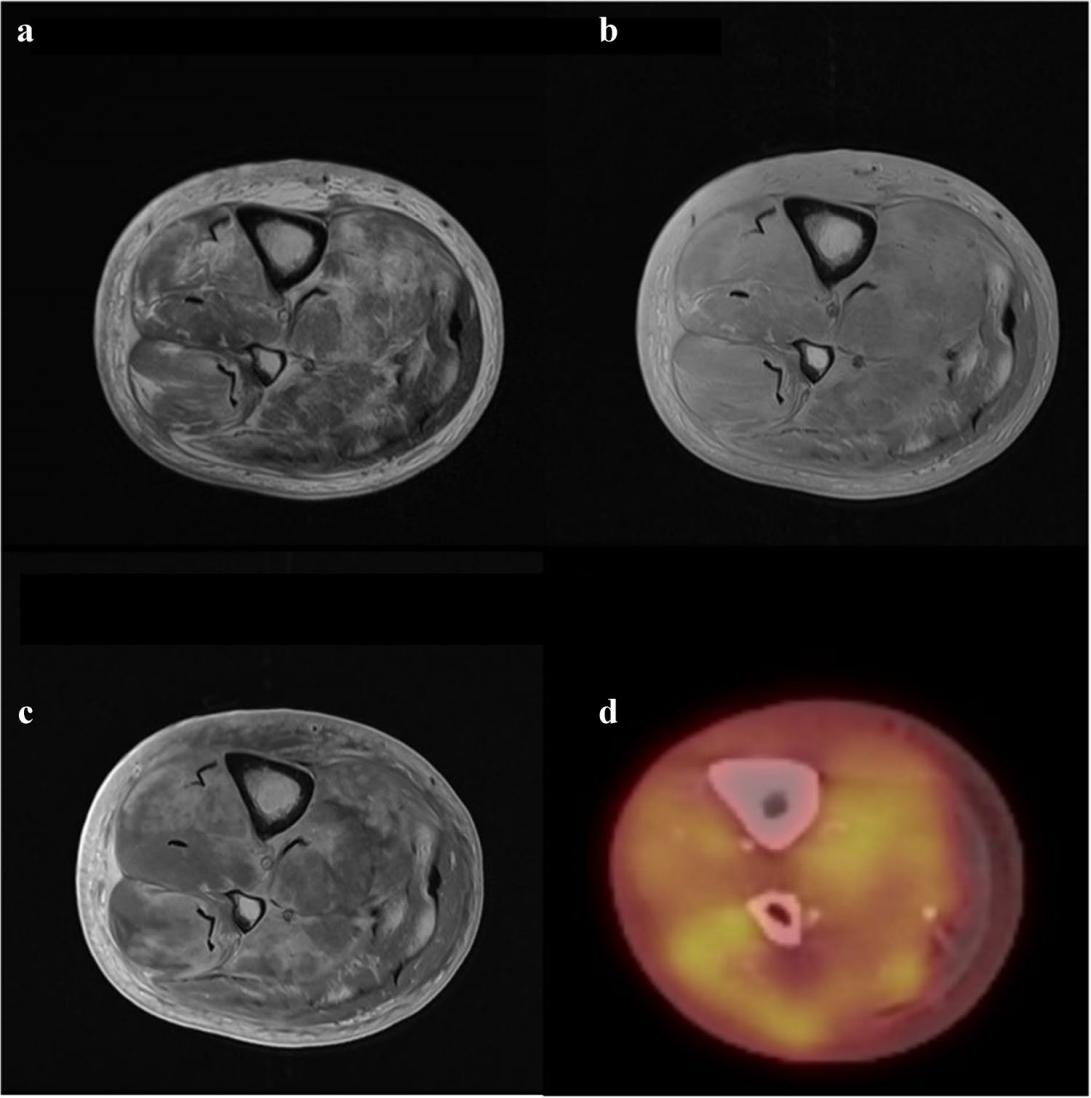
T2-weighted (**a**), PD-weighted (**b**), and contrast-enhanced T1-weighted (**c**) MRI images, illustrating a diffuse abnormal signal intensity in all muscles of the lower leg with band-like appearances at the periphery of the involved muscles. Additionally, there is a diffuse subcutaneous infiltration and thickening of the cutis. The fused PET\CT image (**d**) reveals increased FDG uptake in the same muscles, once again exhibiting a band-like peripheral orientation

**Fig. 2:**
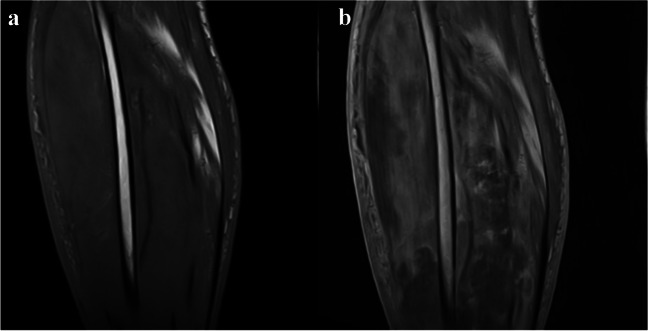
Pre-contrast (**a**) and post-contrast (**b**) T1-weighted MRI images, depicting band-like enhancement at the periphery of the muscles involved in the lower leg

**Fig. 3:**
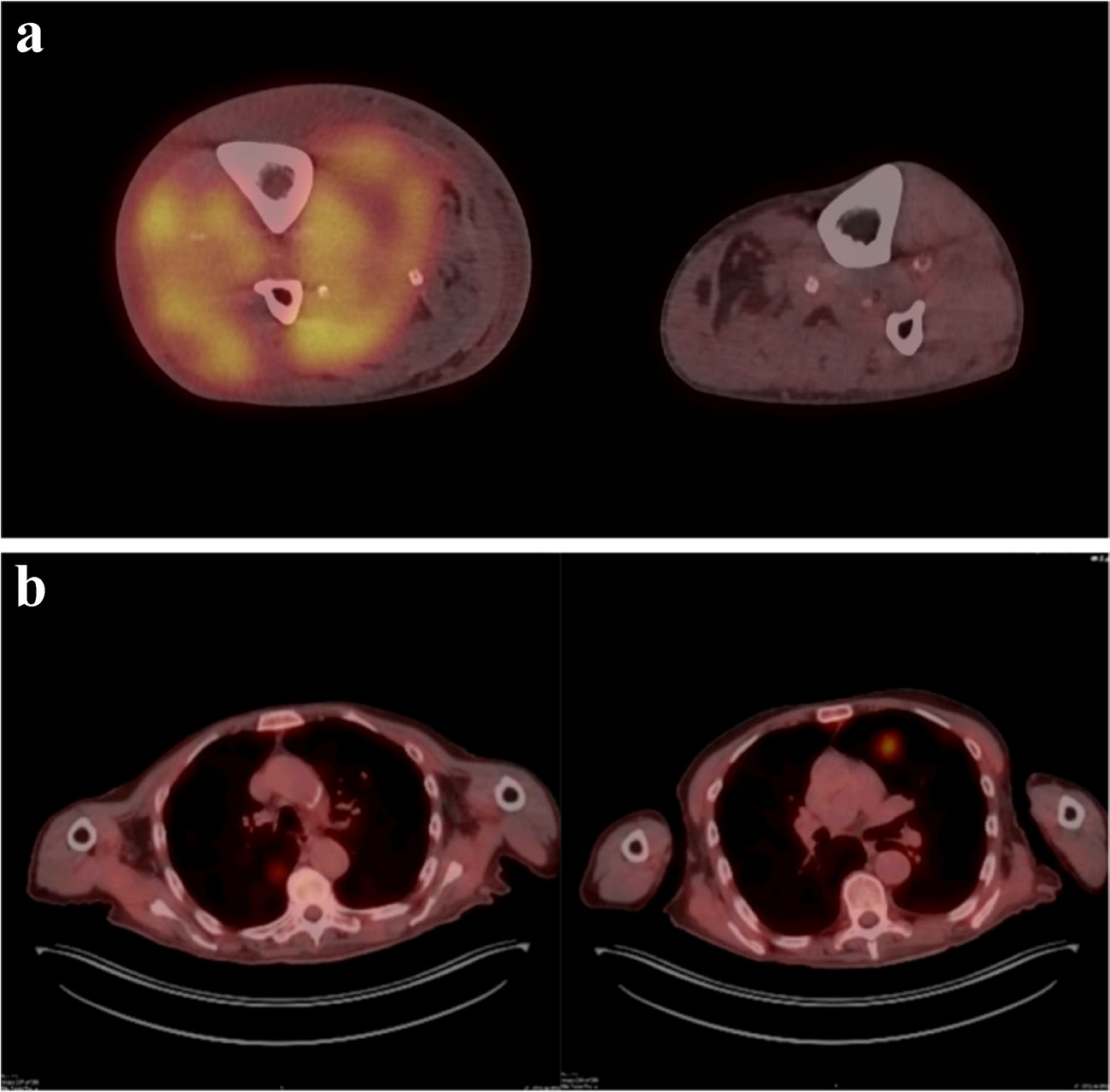
**a** (top) Fused FDG PET\CT images of the lower legs in the axial plane, revealing a noticeable difference in size between. There is significant swelling in the right lower leg, accompanied by increased FDG uptake in both the affected muscles and subcutaneous tissues. **b** (bottom) Fused FDG PET\CT images of the lung showing multiple lung lesions

Two weeks later, an MRI of the right leg revealed diffuse heterogeneous swelling and necrosis affecting multiple muscle compartments in the lower leg. Peripheral band-like abnormal hyper-intense T2-weighted signal intensity and band-like peripheral enhancement on contrast-enhanced T1-weighted images were observed. Additionally, there was associated subcutaneous stranding and skin thickening (Figs. 1 and 2). No evidence of focal masses was detected. A whole-body FDG positron emission tomography/computed tomography (PET/CT) scan showed elevated FDG uptake in the right lower leg, displaying a band-like appearance with heightened uptake in the peripheral region (Fig. 3a). Multiple FDG-positive lung lesions were observed, raising suspicion of metastatic disease (Fig. 3b).

The differential diagnosis included myositis/myonecrosis, infectious causes, and diffuse infiltrating malignancies such as lymphoma. This prompted the consideration of a muscle biopsy. The pathological results from the biopsy of the right gastrocnemius muscle showed predominantly necrotic tissue, making it impossible to make a diagnosis.

Based on the clinical presentation and presence of lung masses, the patient was considered to have paraneoplastic myositis/myonecrosis. Laboratory tests were performed to assess serum antibodies for myositis and creatine-phosphokinase. These were negative, making myositis less likely. Unfortunately, the patient’s condition deteriorated rapidly, resulting in a rescue amputation. At that point, with a septic profile and unknown biopsy results, it was hypothesized pyrogen, paraneoplastic myositis of unknown primary or 4-compartment muscle necrosis due to a neglected chronic compartment syndrome. The whole lower leg was avital with sensory and motor deficits.

**Fig. 4:**
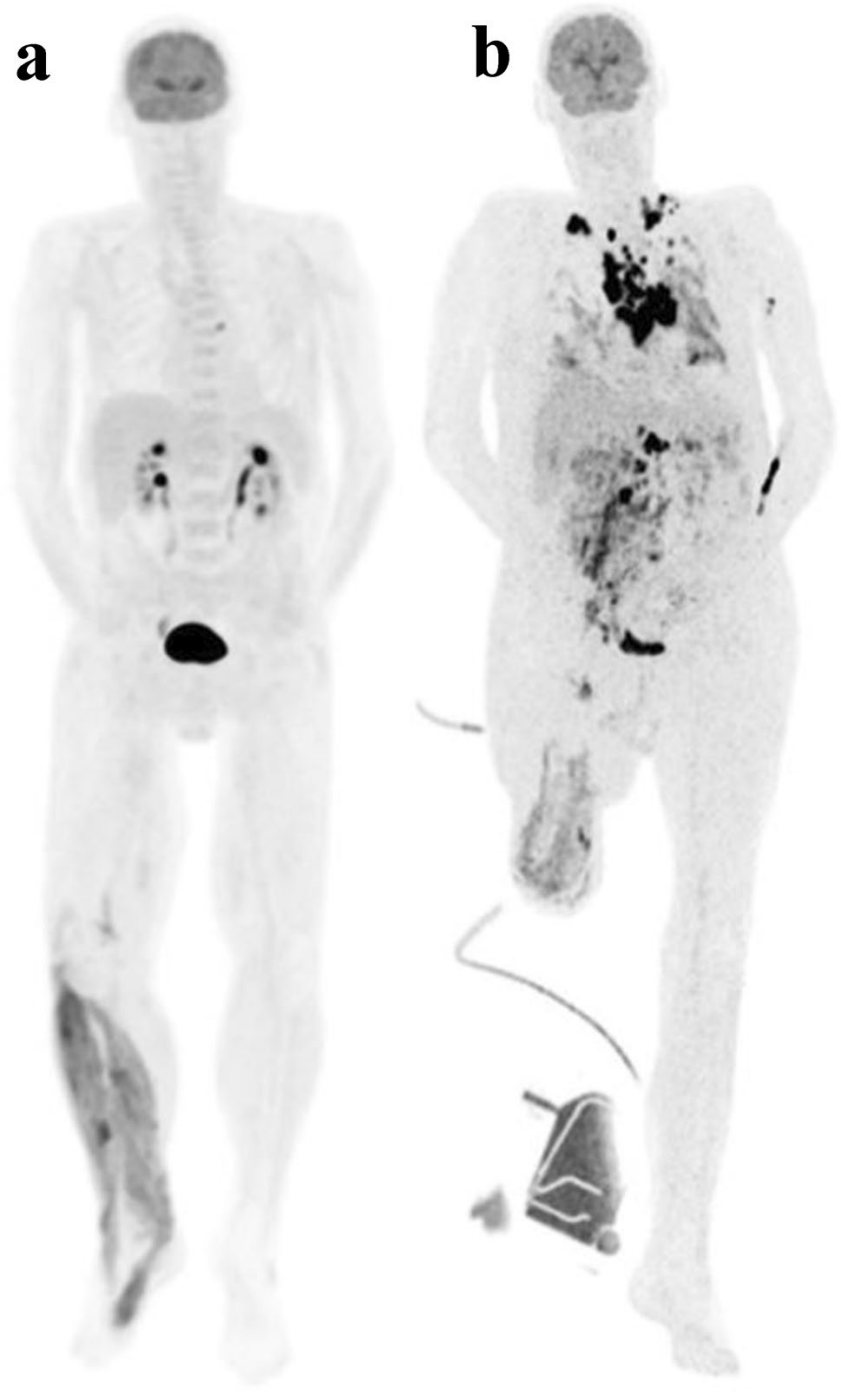
Pre-amputation (**a**) and post-amputation (**b**) maximal intensity projection of FDG uptake. Within just 1 month, there is a pronounced progression of the disease, evidenced by a robust increase in FDG uptake in the pulmonary, lymph nodes, peritoneal, and muscle regions

A post-amputation FDG PET-CT scan demonstrated a substantial progression of the disease, characterized by increased FDG uptake in multiple pulmonary masses, thoracic and abdominal lymph nodes, and diffuse heightened peritoneal FDG uptake (Fig. 4). A lung biopsy was conducted. The patient’s condition deteriorated rapidly, and he died within days.

**Fig. 5:**
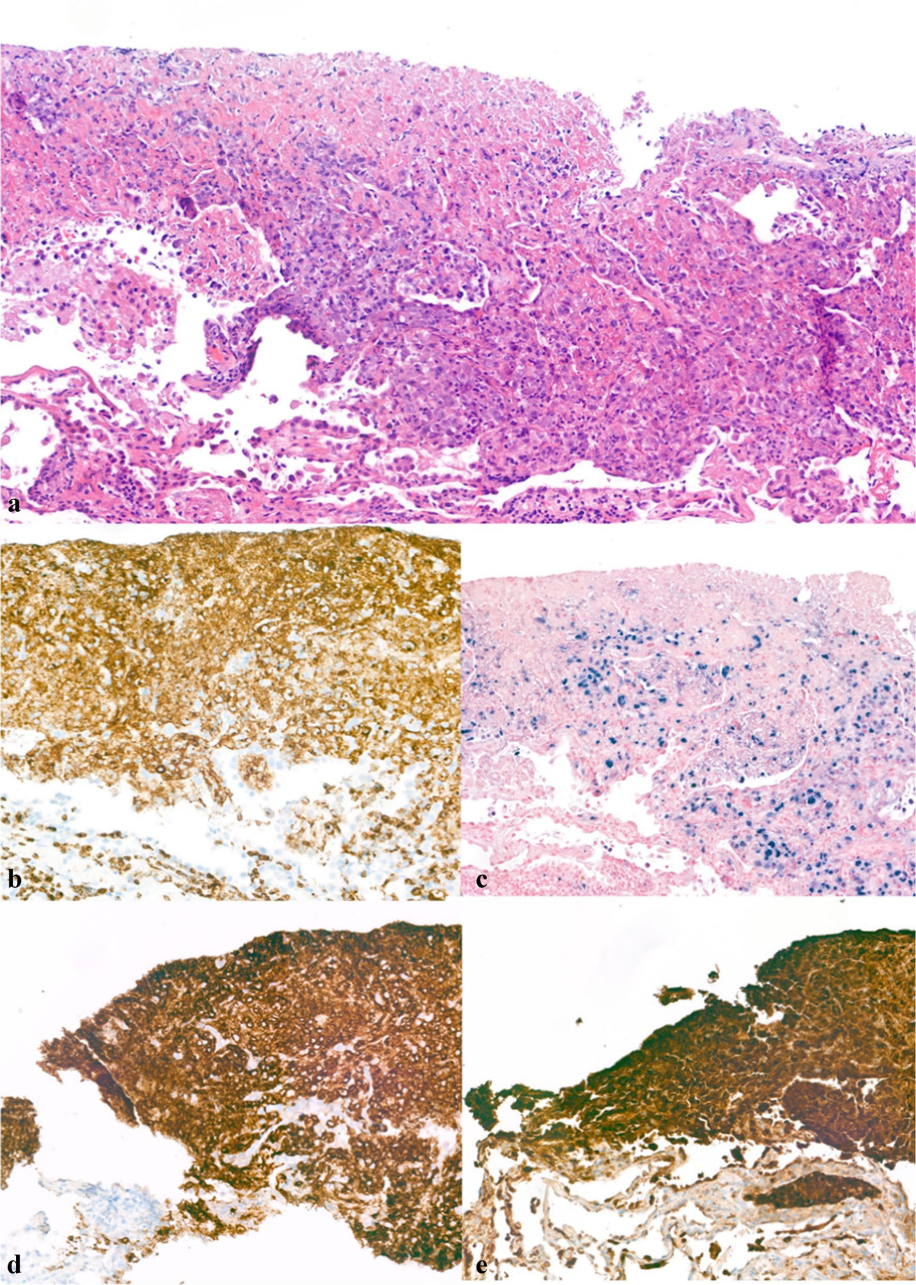
Histological slides of the lung lesion biopsy. The **a** HE staining shows the lesional cells in the mid-right with large, irregular nuclei, positive for **b** CD2, **c** EBER, **d** CD30, and **e** granzyme B staining

Histopathology from a lung sample showed extensive necrosis and an infiltrate of dissociative cells with large nuclei and prominent nucleoli. Some small lymphocytes were present. The atypical population was EBER (Epstein-Barr virus-encoded small RNA’s)) and CD30 positive and showed positivity for some T cell markers (CD2, CD3, and CD7), but also the NK marker granzyme B (Fig. 5). Ki67 showed a high proliferation activity. This population was retrospectively also found in the amputated leg. With this immunohistochemical profile, the definitive diagnosis of extra-nodal NK/T cell lymphoma was established.

## Discussion

Muscular involvement is rarely found in NK/T-cell lymphomas. The differential diagnosis can be challenging, including conditions such as immune myositis, pyomyositis, and diffuse infiltrative processes. In a recent literature review (Table 1), seven cases of extranodal NK/T cell lymphoma were described with an initial presentation of myopathic symptoms. These cases were predominantly misclassified as myositis. Among these, five cases exhibited a more generalized, symmetric muscle involvement [7–11] while only two cases were characterized as granulomatous and phlegmonous myositis, featured an asymmetric and mono-compartmental [12, 13]; all reported cases were correlated with Epstein-Barr virus (EBV) infection.

**Table 1:** Previous cases of natural killer/T cell lymphoma with muscular involvement

Literature references	Muscle involvement	EBV test
Liu et al. (7)	Generalized	+
Shi et al. (8)	Generalized	+
Hashimoto et al. (9)	Generalized	+
Kawaguchi et al. (10)	Generalized	+
Chan et al. (11)	Generalized	+
Min et al. (12)	Localized	+
Yang et al. (13)	Localized	+

In our case, radiological and histological findings strongly suggested the diagnosis of necrotizing myopathy, despite the infrequent presentation of asymmetric involvement. Notably, the presence of lung lesions on PET-CT led to the initial misdiagnosis of a paraneoplastic process. Pyomyositis is typically characterized by the presence of intramuscular abscesses, while the diffuse infiltrative process, as seen in lymphomas, typically presents with a mass-like involvement, which was not present.

Furthermore, following the inconclusive results of the US, which did not reveal any signs of DVT, no additional investigation was conducted to determine the cause of the pain and swelling in the leg. Instead, the leg was simply bandaged daily by nurses. The absence of this delay could have potentially led to an earlier diagnosis, possibly as much as 2 months sooner.

In managing a case potentially misdiagnosed as myositis, leading to a dramatic presentation indicative of extranodal NK/T cell lymphoma, a comprehensive and multidisciplinary strategy is crucial. This strategy should prioritize lymphoma in the differential diagnoses for patients presenting with systemic symptoms, muscle swelling, and aspecific imaging patterns especially when common conditions have been ruled out. Employing advanced imaging techniques like MRI and FDG PET/CT scans, in conjunction with tissue biopsies, is essential for accurate diagnosis. Due to the disease’s aggressive nature, it is imperative to quickly start a multimodal treatment approach.
